# Bleeding events in bevacizumab-treated cancer patients who received full-dose anticoagulation and remained on study

**DOI:** 10.1038/sj.bjc.6606074

**Published:** 2011-01-18

**Authors:** N B Leighl, J Bennouna, J Yi, N Moore, J Hambleton, H Hurwitz

**Affiliations:** 1 Division of Medical Oncology, Princess Margaret Hospital, 5th Floor Room 105, 610 University Avenue, Toronto, Ontario M5G 2M9, Canada; 2 Division of Medical Oncology, Centre René Gauducheau, Nantes Saint-Herblain, France; 3 Genentech, Inc., South San Francisco, CA, USA; 4 Hoffman-La Roche, Inc., Basel, Switzerland; 5 Division of Medical Oncology, Duke University School of Medicine, Durham, NC, USA

**Keywords:** bevacizumab, non-small cell lung cancer, colorectal cancer, anticoagulation, haemorrhage, safety

## Abstract

**Background:**

Bevacizumab provides clinical benefit in multiple solid tumours, but is associated with some increase in bleeding risk. Thrombotic events necessitating therapeutic anticoagulation (TA) are common in cancer. This report describes the safety of concurrent bevacizumab and TA in three large placebo-controlled clinical studies.

**Methods:**

Study 1 (metastatic colorectal cancer (mCRC)), study 2 (mCRC), and study 3 (advanced non-small cell lung cancer) were blinded phase III studies. Eligibility criteria excluded patients on TA. Patients on protocol treatment who developed thrombotic events requiring TA were permitted to continue bevacizumab or placebo under specified conditions. Adverse events in patients who received bevacizumab and TA concurrently were assessed using the NCI–CTCAE scale.

**Results:**

While experience is limited, venous thrombotic events were the most common reason for TA initiation in the three studies. Severe bleeding event rates for patients receiving TA in the bevacizumab-treated groups were similar in frequency to the placebo groups, ranging from 0 to 8% or 0 to 67 events per 100 patient-years. No severe pulmonary bleeding was reported in any of the TA-treated populations.

**Conclusions:**

These data suggest that bevacizumab did not increase the risk of severe bleeding in cancer patients who received TA.

Bevacizumab is a monoclonal antibody targeting vascular endothelial growth factor, a key regulator of angiogenesis. When administered with chemotherapy, bevacizumab has been shown to improve survival, progression-free survival (PFS) and response rates (RRs) in metastatic colorectal cancer (mCRC) (Hurwitz *et al*, 2004) and advanced non-small cell lung cancer (NSCLC) (Sandler *et al*, 2006). Additional phase II and III studies have reported statistically and clinically significant improvements in PFS and/or RR for mCRC (Kabbinavar *et al*, 2005; Saltz *et al*, 2008), NSCLC (Reck *et al*, 2009), breast cancer (Miller *et al*, 2007; Fumoleau *et al*, 2008), renal cell carcinoma (Escudier *et al*, 2007; Rini *et al*, 2008), and glioblastoma multiforme (Vredenburgh *et al*, 2007).

Bevacizumab use has been associated with an increase in the risk of bleeding. Although bleeding events involved typically minor epistaxis and other self-limited mucosal bleeding, severe (grade ⩾3) bleeding events have also been reported (Hurwitz *et al*, 2004; Kabbinavar *et al*, 2005; Sandler *et al*, 2006; Escudier *et al*, 2007; Miller *et al*, 2007; Vredenburgh *et al*, 2007; Fumoleau *et al*, 2008; Rini *et al*, 2008; Saltz *et al*, 2008; Reck *et al*, 2009). In NSCLC, a randomised phase II study of bevacizumab in combination with carboplatin and paclitaxel reported severe or fatal pulmonary haemorrhage (PH) in 9% of bevacizumab-treated patients (Johnson *et al*, 2004). On review, it was noted that PH occurred in 4 of 13 patients (31%) with squamous NSCLC histology but in only 2 of 54 patients (4%) with non-squamous histology. This led to the exclusion of patients with predominantly squamous cell histology from subsequent pivotal phase III studies of bevacizumab in NSCLC. The reported incidences of severe PH in the bevacizumab-treated groups in two large phase III NSCLC studies were 1.9 and 1.2% (Sandler *et al*, 2006; Reck *et al*, 2009). In NSCLC, as well as other tumour types, small increases in the incidence of severe non-pulmonary bleeding have also been reported, primarily involving the gastrointestinal (GI) and genitourinary tract, and the central nervous system (CNS) (Johnson *et al*, 2004; Sandler *et al*, 2006).

Life-threatening venous thromboembolic events (VTEs) including deep vein thrombosis (DVT) and pulmonary embolism (PE) are common in cancer patients and represent a leading cause of morbidity and mortality in outpatients receiving cancer chemotherapy (Heit *et al*, 2000; Khorana *et al*, 2007). Treatment of VTEs with therapeutic anticoagulation (TA), using unfractionated or low molecular-weight heparin (LMWH) and/or vitamin K antagonists, such as warfarin, is the preferred approach for management (Gitter *et al*, 1995; Hutten *et al*, 2000; Deitcher *et al*, 2006; Lyman *et al*, 2007). However, bleeding complications associated with anticoagulation in cancer patients, irrespective of the anticoagulant used, are more frequent than in non-cancer patients receiving TAs (Gitter *et al*, 1995; Palareti *et al*, 1996; Hutten *et al*, 2000; Lee *et al*, 2003; Deitcher *et al*, 2006). A study by Hutten *et al* (2000) showed that the rate of major bleeding was 13.3 events per 100 person-years for patients with malignancy, compared with 0.3 to 1.1 events per 100 patient-years in patients receiving TA who do not have underlying malignant disease. In the CLOT study, cancer patients who developed their first DVT were randomised to LMWH followed by an oral vitamin K antagonist *vs* continued LMWH (Lee *et al*, 2003). Major bleeding, (defined as bleeding resulting in death, transfusion of ⩾2 units of blood, a ⩾2.0 g dl^–1^ fall in haemoglobin, or bleeding in a critical location (intracranial, intraocular, intraspinal, retroperitoneal, or pericardial)), occurred in 4% of patients receiving the oral vitamin K antagonist, compared with 6% of patients continuing on LMWH, after 6 months of anticoagulation. Important risk factors for severe bleeding on TA include age and comorbidities (Palareti *et al*, 1996).

Given the frequency of thromboembolic disease in cancer patients and the subsequent requirement for TA, it is not unexpected that many patients being considered for, or actively receiving, bevacizumab therapy may also require TA treatment. Currently, there is limited information available about the safety of combining bevacizumab and TA therapy. We sought to describe the bleeding risk in patients receiving TA and bevacizumab in a retrospective analysis of three large, placebo-controlled studies.

## Materials and Methods

### Selection of studies

The three randomised, placebo-controlled clinical trials analysed in this report each permitted co-administration of study drug (bevacizumab or placebo) and TA. Of the many trials that have been conducted for bevacizumab treatment of solid tumour cancers, as of 31 March 2008, these three trials (two for treatment of mCRC (Hurwitz *et al*, 2004; Saltz *et al*, 2008) and one for NSCLC (Reck *et al*, 2009) have provided the most detailed information on concomitant use of medication, including anticoagulants. The selection of anticoagulation agents in all three studies was at the discretion of treating physicians.

### Study details

All patients participating in the three trials were enrolled after providing informed consent. Human investigations were performed after institutional review board approval, in accord with an assurance approved by the US Department of Health and Human Services. Details of study design and eligibility have been previously described (Hurwitz *et al*, 2004; Saltz *et al*, 2008; Reck *et al*, 2009). Patients with a recent history (6 months to 1 year) of cardiovascular disease were excluded from the trials, as were those treated with TA at baseline.

Patients in all three studies provided written informed consent according to federal and institutional guidelines before study procedures began. Human investigations were performed after ethical approval by a human investigations committee and in accordance with an assurance filed with and approved by the Department of Health and Human Services.

#### Study 1.

In this study, 789 patients with previously untreated CRC, received irinotecan, leucovorin, fluorouracil chemotherapy plus bevacizumab or placebo (5 mg kg^–1^ every 2 weeks) (Hurwitz *et al*, 2004). In the original protocol, current or recent (within 10 days before day 0) use of full-dose oral or parenteral anticoagulants was not permitted at study entry, and patients who developed a thrombotic event requiring TA were discontinued from study therapy. Persistent (⩾3 weeks) grade 3 or 4 AEs, or any significant AE that compromised the subject's ability to participate in the study, were also cause for study discontinuation. However, as some patients were judged by the respective investigators to have benefited from study treatment, protocol exceptions were granted to permit resumption of study treatment after TA, provided there were no additional risk factors for bleeding. An analysis of bleeding event rates in those patients showed no excess bleeding risk, and the study was amended to allow continued participation on TA if the patient had not been unblinded. Study 1 was conducted predominantly in the United States, between 2000 and 2003.

#### Study 2.

This was a 2 × 2 factorial, randomised, multi-centre, multi-national phase III study, with four treatment arms (FOLFOX+bevacizumab (5 mg kg^–1^ every 2 weeks), FOLFOX+placebo, XELOX+bevacizumab (7.5 mg kg^–1^ every 3 weeks), and XELOX+placebo) (Saltz *et al*, 2008), in which 1369 patients with inoperable mCRC, who had not previously received systemic treatment for metastatic disease, were treated with bevacizumab or placebo. Study 2 was conducted predominantly outside the United States between 2004 and 2005. Patients with current or recent use of anticoagulants (within 10 days before study treatment) were excluded from study entry, except where anticoagulants were used for maintenance of preexisting IV catheters. Standard discontinuation criteria applied in this study: investigators could also withdraw patients from the study in the event of intercurrent illness, AEs, treatment failure, protocol violations, administrative reasons, or other reasons.

#### Study 3.

Patients with inoperable stage IIIb, IV, or recurrent non-squamous NSCLC (*N*=1043) were treated with gemcitabine/cisplatin first-line chemotherapy plus bevacizumab or placebo (Reck *et al*, 2009). Two bevacizumab doses were tested, 7.5 and 15 mg kg^–1^, every 3 weeks, and were pooled for this analysis. Study 3 was conducted exclusively outside of the United States, between 2005 and 2006. Patients with current or recent therapeutic use of full-dose anticoagulants or thrombolytic agents (within 10 days before study treatment) were excluded from the study. Standard study discontinuation criteria were described in the study protocol (see Study 2 above).

### Data collection on TA and adverse events

Patients in this report were identified as those receiving an anticoagulant medication concurrently with study drug following a VTE during the study. Patients permitted to continue on bevacizumab and TA were required to have no evidence of haemorrhage during study; were required to be on 2–3 weeks of TA, with INR within target range for warfarin and on a stable dose of anticoagulant; and, for studies 1 and 2, did not have any evidence of tumour invading or abutting major blood vessels, by CT scan. All studies excluded patients requiring TA or aspirin >325 mg day^–1^ at study entry and patients with known bleeding diathesis. None of the studies collected specific information regarding dosage, frequency, or duration of anticoagulant agent, and only study 3 collected information about anticoagulant indication (prophylactic or therapeutic). Patients who experienced a VTE were permitted to resume bevacizumab or placebo after TA was established with a stable dose of an anticoagulant. Those who developed arterial thrombosis were required to discontinue bevacizumab/placebo treatment. None of the studies permitted crossover to bevacizumab from the placebo group.

Adverse events, including bleeding severity, were assessed using the NCI–CTCAE scale (version 2.0 for study 1; version 3.0 for studies 2 and 3) (Trotti *et al*, 2000, 2003). Information on bleeding AEs of all grades was collected in studies 2 and 3. In study 1, however, information on all grades of AEs was only collected until a pre-planned safety interim analysis, after approximately one-third of the total sample size was enrolled; after this point, only grade ⩾3 AE's were collected. NCI–CTC grade ⩾3 events are defined as severe adverse events (SAEs). Thrombotic complications included DVT, PE, and arterial ischaemic events. By definition, DVT and PE requiring anticoagulation are defined as grade 3 or 4, and are therefore reported for all patients in study 1. All patients who received TA and study treatment concurrently, following a treatment emergent VTE, were included in this analysis. Treatment emergent VTE was defined as VTE with onset on or after the start of protocol treatment.

In study 1, AEs were collected for 14 days after the final dose of study drug during first-line therapy. For patients who went on to receive BV as second-line therapy as a single agent or in combination with chemotherapy, the serious AEs were collected at cycle day 0, 7, 14, 21, 28 and 35. The other two studies collected information on AEs up to 28 days after each patient ended the study medication.

All bleeding SAEs were individually reviewed, with the incidence defined by the number of patients with at least one AE as indicated, and by highest grade as indicated. The number of person-years of observation is defined as the sum of the time from the first day on which TA was received following a VTE to the date of the first bleeding AE that occurred after first use of anticoagulant medication. Patients without a bleeding event after anticoagulant use were censored at the last date of TA or study drug use, whichever occurred first.

## Results

### Thrombotic adverse events and treatment

Treatment emergent grades 3–4 VTEs occurred at rates ranging between 5.0 and 15.3% in the placebo- and bevacizumab-treated groups in the three studies (Table 1). The incidence of these events in each study was no higher for patients receiving bevacizumab than for those receiving control.

### Anticoagulation agents

In study 1, warfarin was the TA used by the vast majority (∼95%) of patients who received TA while on study drug. In study 2, ∼40% of patients who received TA while on study drug used warfarin and the remainder used LMWH. In study 3, ∼35% of patients receiving TA while on study drug received warfarin, with the remainder using LMWH.

### Study treatment status following start of TA

There were a total of 194 patients in the three trials who received concurrent study treatment and TA treatment.

**Figure 1:**
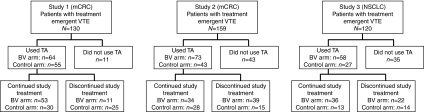
Study flow charts for patients with treatment emergent VTE.

In study 1, of the 64 patients in the bevacizumab group who received TA for thrombosis, 53 (83%) continued TA and study treatment concomitantly for a median of 27 weeks. Of the 55 patients in the placebo group who started TA for a thrombotic event, 30 (55%) continued TA and study treatment for a median of 19 weeks (Figure 1).

In study 2, of the 73 patients in the bevacizumab-containing group who received anticoagulation treatment following a treatment emergent VTE event, 34 (47%) continued study treatment and concurrent anticoagulants for a median of 14 weeks (Figure 1). Of the 43 patients in the placebo group who received anticoagulation treatment following a VTE event, 28 (65%) continued study treatment and concurrent anticoagulants for a median of 19 weeks.

In study 3, of the 58 bevacizumab-treated patients who started TA for a thrombotic event, 36 (62%) continued study treatment plus concurrent TA for a median of 8 weeks (Figure 1). Of the 27 patients in the placebo group who began TA, 13 (48%) continued study treatment + TA for a median of 2 weeks.

### Bleeding adverse events in the TA-treated population

In the three studies analysed in this report, the overall rates of severe bleeding for all patients in the control *vs* the bevacizumab groups were: 2.5 *vs* 3.3% in study 1, 1.2 *vs* 1.9% in study 2, and 1.2 *vs* 3.5% in study 3, consistent with the small increase in risk typically reported in controlled bevacizumab studies. The rates of all bleeding events (any grade) in patients on TA were assessed in studies 2 and 3 (Table 2). Rates of severe (grade ⩾3) bleeding events were assessed for all three studies (Table 2), and were similar among the control/placebo- and bevacizumab-treated groups: 7 *vs* 4% in study 1, 0 *vs* 3% in study 2, and 8 *vs* 6% in study 3, respectively (Table 2). There were three severe bleeding events in the placebo groups (GI bleeding, CNS bleeding, and bleeding not otherwise specified) and five in the bevacizumab groups (rectal bleeding, retroperitoneal bleeding, CNS bleeding, and two epistaxis events). Among the eight patients who experienced severe bleeding on TA, two patients, both of whom received bevacizumab, had concomitant thrombocytopenia (grade 1 thrombocytopenia in study 1, grade 4 thrombocytopenia in study 3). The estimated overall risk of severe bleeding was 4.1% in the pooled bevacizumab group, and 4.2% in the pooled control group.

As bevacizumab provided significant improvement in time to disease progression in all of these studies, we corrected for differences in both time to bleeding event and overall observation time by calculating rates of severe (grade⩾3) bleeding events per 100 person-years. These rates, ranging from 0 to 67 events per 100 person-years, were similar for control and bevacizumab groups. By combining the relatively small number of bleeding events from all three studies, the estimated overall risk of severe bleeding was 9.0 per 100 patient-years in the pooled bevacizumab group, and 10.5 per 100 patient-years in the pooled control group.

There were no reports of severe (grade ⩾3) PH among any TA-treated patients in the bevacizumab treatment groups in these studies. No fatal bleeding events occurred in any TA-treated patient.

## Discussion

In patients with advanced cancer, thrombosis is a common event and a major source of morbidity and mortality. The standard treatment for significant thrombosis is TA, typically starting with heparinoids, such as unfractionated or LMWH, followed by oral vitamin K antagonists or continued heparinoids. Unfortunately, anticoagulation therapy in both cancer and non-cancer populations is often complicated by major bleeding (Gitter *et al*, 1995; Palareti *et al*, 1996; Hutten *et al*, 2000; Lee *et al*, 2003; Deitcher *et al*, 2006). Nevertheless, the risk–benefit assessment for TA usually favours its use because of the immediate life-threatening consequences of thrombosis.

Bevacizumab has been associated with an increased risk of bleeding of all grades, although most bleeding events are mild, self-limited, and frequently mucosal without need for medical intervention or bevacizumab discontinuation (Hurwitz *et al*, 2004; Kabbinavar *et al*, 2005; Sandler *et al*, 2006; Escudier *et al*, 2007; Miller *et al*, 2007; Vredenburgh *et al*, 2007; Fumoleau *et al*, 2008; Rini *et al*, 2008; Saltz *et al*, 2008; Reck *et al*, 2009). An increased risk of severe (grade ⩾3) bleeding has also been reported with bevacizumab treatment. Severe bleeding has occurred at primary sites of malignant disease, such as PH in NSCLC patients and GI bleeding in CRC patients. However, severe bleeding has also been described at sites not primarily involved with cancer, such as GI bleeding in patients without CRC, and CNS bleeding in patients without known brain metastases (Johnson *et al*, 2004; Sandler *et al*, 2006).

The bleeding rates reported in this analysis compare favourably with bleeding risks reported in the literature for cancer patients receiving TA (approximately 13 major bleeding events per 100 patient-years) (Hutten *et al*, 2000). The number of TA patients with severe bleeding events in the three studies analysed in this report precluded a definitive analysis of the relative safety of warfarin *vs* LMWH in combination with bevacizumab. Of the five patients receiving concurrent therapy and experiencing a severe bleeding event in the combined bevacizumab-containing groups, two were receiving heparin and three were receiving warfarin.

Given the uncommon, but potentially life-threatening complication of severe PH in patients with NSCLC receiving bevacizumab, the use of anticoagulation in study 3 is of particular interest. Of the eight grade ⩾3 PH events reported among bevacizumab-treated patients (Reck *et al*, 2009), no PH events were reported in patients receiving TA. Although the number of TA patients in study 3 was relatively small, these findings are reassuring regarding the concomitant use of anticoagulants and bevacizumab in NSCLC.

It should be noted that these analyses were conducted for the subset of patients experiencing a VTE event in these studies. In addition, the study population needed to meet baseline eligibility criteria, which excluded many conditions that may increase bleeding risks. The proportion of patients continuing on study drug and TA following a VTE ranged from 47 to 83%. The reasons that investigators chose to either not initiate TA following a VTE or to not continue study drug therapy following TA were not captured. Possible reasons include: that these patients may have had clinical contraindications to TA, patients could not be successfully anticoagulated, patients showed signs of clinical progression, and/or patients were too ill to continue on study drug therapy. A further limitation of this analysis is that data on the level, frequency, and duration of anticoagulation was not routinely collected in these three studies, and that duration on anticoagulation was relatively short in the NSCLC trial (Reck *et al*, 2009). In addition, dosing information was not always collected for LMWH and other anticoagulants. Therefore, the influence of these factors on bleeding incidence cannot be assessed. The power of this study is also limited by the numbers of patients, the relatively low numbers of bleeding events, and the high rate of discontinuation for patients who developed VTE and received TA. Other studies that included TA for patients treated with bevacizumab could not be analysed in this report, because detailed information about use of anticoagulants was not collected in these other studies. These caveats should be taken into account when attempting to extrapolate these findings broadly.

Analyses from distinct data sets have evaluated the risks of anticoagulation and appear to confirm the relative safety of concurrently administering TA and bevacizumab in cancer patients. BRiTE, a large observational cohort study for mCRC patients receiving bevacizumab-based therapy, enrolled 1953 patients, primarily in the community setting, with 133 (6.8%) receiving concomitant TA at some point during their treatment course (Grothey *et al*, 2008). The observed rate of severe bleeding in this group of patients was 6.0% (Flynn *et al*, 2008). This estimate of risk is consistent with the results of this report and also consistent with the incidence of severe bleeding in on TA who are cancer patients not receiving bevacizumab. In addition, results from a large randomised study evaluating bevacizumab in high-grade glioma have recently been reported. Of the 163 patients enrolled and treated with bevacizumab, 48 (29.4%) received concomitant TA, and 2 (4%) of these patients experienced a severe bleeding event (1 patient had grade 3 menorrhagia and another developed grade 3 tumour-associated CNS haemorrhage) (Friedman *et al*, 2009).

In the phase IV international SAiL study of bevacizumab in 1065 NSCLC patients, there were a total of 19 bleeding events in 15 of 87 patients (17.2% none grade ⩾3) receiving anticoagulants (mostly LMWH) at baseline, compared with 227 bleeding events in 181 patients (17.0%) for the entire treated population (Griesinger *et al*, 2008). Although 15% of patients received concomitant anticoagulation therapy at some point during SAiL, the overall incidence of grade ⩾3 bleeding was low (4%) (Crino *et al*, 2010). These data suggested that concomitant use of anticoagulation and bevacizumab-based therapy was feasible and did not substantially increase the risk of clinically significant bleeding in NSCLC. Preliminary results from the bevacizumab treatment registry ARIES have shown no incidence of PH among 65 patients with NSCLC on TA; future analyses from this registry will assess the overall bleeding risks for patients on TA (Kumar *et al*, 2010).

Bevacizumab is an important therapeutic option for many patients with common malignancies who will frequently require TA. This report provides important information to clinicians for assessing individual risk–benefit decisions for patients requiring TA in the setting of bevacizumab-containing therapy. Although experience is limited, these results suggest that combining bevacizumab with TA does not appreciably increase the risk of bleeding above the risk of bleeding expected from TA alone.

## Figures and Tables

**Table 1:** Overall incidence of thrombotic and bleeding adverse events (treated patients)

	**Study 1 (mCRC)^a^**		**Study 2 (mCRC)**		**Study 3 (NSCLC)^b^**	
**Adverse event**	**IFL/placebo (*n*=397)**	**IFL/BV (*n*=392)**	**FOLFOX-4/XELOX/ placebo (*n*=675)**	**FOLFOX-4/XELOX/ BV (*N*=694)**	**CG/placebo (*n*=327)**	**CG/BV (*n*=659)^b^**
*Patients with any grade events, n* (%)						
Arterial thrombosis	5 (1.3)	14 (3.6)	10 (1.5)	17 (2.4)	18 (5.5)	25 (3.8)
Venous thrombosis	62 (15.6)	68 (17.3)	65 (9.6)	94 (13.5)	35 (10.7)	85 (12.9)
Bleeding/haemorrhage	NA^a^	NA^a^	175 (25.9)	212 (30.5)	67 (20.5)	239 (36.3)
						
*Patients with grade 3/4 events, n* (%)						
Venous thrombosis	55 (13.8)	60 (15.3)	34 (5.0)	56 (8.1)	21 (6.4)	47 (7.1)
Deep vein thrombosis	27 (6.8)	35 (8.9)	10 (1.5)	21 (3.0)	5 (1.5)	13 (2.0)
Pulmonary embolus	20 (5.0)	15 (3.8)	7 (1.0)	18 (2.6)	10 (3.1)	26 (3.9)
Bleeding/haemorrhage	10 (2.5)	13 (3.3)	8 (1.2)	13 (1.9)	4 (1.2)	23 (3.5)

Abbreviations: BV=bevacizumab; CG=cisplatin+gemcitabine; FOLFOX-4=oxaliplatin, folinic acid and 5-fluorouracil; IFL=irinotecan/5-fluorouracil/leukovorin; mCRC=metastatic colorectal cancer; NA=not applicable; NSCLC=non-small cell lung cancer; XELOX=capecitabine plus oxaliplatin.

a In study 1, only grades 3–4 bleeding events were uniformly collected.

b The bevacizumab dose groups in study 3 (7.5 and 15 mg kg^–1^ every 3 weeks) were pooled.

**Table 2:** Incidence of bleeding AEs in patients receiving TA and concurrent study treatment^a^

	**Study 1 (mCRC)^b^**		**Study 2 (mCRC)**		**Study 3 (NSCLC)^c^**		**Pooled studies^d^**	
**Study 1 (mCRC)^b^** **Placebo (*n*=30)**	**IFL/placebo (*n*=30)**	**IFL/BV (*n*=53)**	**FOLFOX-4/XELOX/placebo (*n*=28)**	**FOLFOX-4/XELOX/BV(*N*=34)**	**CG/placebo (*n*=13)**	**CG/BV (*n*=36)**	**Pooled placebo groups (*n*=71)**	**Pooled bevacizumab groups (*n*=123)**
*Patients with bleeding AEs n*(%)								
All grades	NA^b^	NA^b^	9 (32)	11 (32)	5 (38)	12 (33)	14 (34)	23 (33)
Epistaxis	NA^b^	NA^b^	6 (21)	10 (29)	2 (15)	11 (31)	8 (20)	21 (30)
Pulmonary haemorrhage/ haemoptysis	NA^b^	NA^b^	0	1 (2.9)	1 (8)	1 (3)	1 (2)	2 (3)
Other AEs	NA^b^	NA^b^	7 (25)^e^	4 (12)^e^	3 (23)^f^	3 (8)^f^	10 (24)	7 (10)
Grades 3–4^g^	2 (7)	2 (4)	0	1 (3)	1 (8)	2 (6)	3 (4)	5 (4)
Epistaxis	0	0	0	0	0	2 (6)	0	2 (2)
Pulmonary haemorrhage/ haemoptysis	0	0	0	0	0	0	0	0
GI bleeding	1 (3)	0	0	0	0	0	1 (1)	0
CNS bleeding	0	1 (2)	0	0	1 (8)	0	1 (1)	1 (1)
Other grades 3–4 AEs^h^	1 (3)	1 (2)	0	1 (3)	0	0	1 (1)	2 (2)
Rate of severe (grade ⩾3) bleeding per 100 persons-years	13	6	0	9	67	20	10.5	9.0

Abbreviations: AE=adverse event; BV=bevacizumab; CG=cisplatin+gemcitabine; CNS=central nervous system; FOLFOX-4=oxaliplatin, folinic acid, 5-fluorouracil; GI=gastrointestinal; IFL=irinotecan+5-fluorouracil+leukovoran; mCRC=metastatic colorectal cancer; NA=not applicable; NSCLC=non-small cell lung cancer; TA=therapeutic anticoagulation; XELOX=capecitabine+oxaliplatin.

a For patients with more than one event of the same type, the highest grade event is reported.

b Only severe bleeding events were captured in study 1 (grades 3–4).

c The bevacizumab dose groups in study 3 (7.5 and 15 mg kg^–1^ every 3 weeks) were pooled in this analysis.

d The pooled studies analysis includes studies 2 and 3 for all grade bleeding, and studies 1–3 for grades 3–5 bleeding.

e The other bleeding events in the study 2 placebo cohort were: one grade 2 haematuria, two grade 1 gingival bleeding events, and one each of grade 1 rectal haemorrhage, urinary tract haemorrhage, haemorrhoidal haemorrhage and melaena. The other bleeding events in the cohort that received bevacizumab were: two Grade 1 haematuria events, a grade 3 rectal haemorrhage and a grade 1 rectal haemorrhage.

f The other bleeding events in study 3 were two petechiae events (one patient in each treatment cohort), two GI bleeds (one in each cohort) and two CNS bleeds (one in each cohort).

g No grade 5 AEs were observed among TA-treated patients in these studies.

h The other grades 3–4 bleeding events in study 1 were grade 3 haemorrhage (not otherwise specified) in the IFL/placebo cohort and grade 3 retroperitoneal haemorrhage in the IFL/BV group. In study 2, there was a grade 3 rectal haemorrhage in the cohort that received bevacizumab.
